# Mediators of the Relationship Between Functional Limitations and Loneliness Among Homebound African Americans

**DOI:** 10.1093/geroni/igaa057.941

**Published:** 2020-12-16

**Authors:** Maritza Dowling, Hiroko Dodge, Antonio Puente, Beverly Lunsford

**Affiliations:** 1 George Washington University - School of Nursing, Washington, District of Columbia, United States; 2 Pregon Health & Science University, Portland, Oregon, United States; 3 School of Medicine & Health Sciences, Washington, District of Columbia, United States; 4 George Washington University-School of Nursing, Washington, District of Columbia, United States

## Abstract

Physical/functional limitations are known to lead to loneliness, but there is little research on protective factors that may mediate their adverse association, particularly in vulnerable populations. This study used cross-sectional survey data from 147 (aged 58-90 years; 75% females) predominantly low-income African American homebound community dwellers to investigate the role of resilience and social connectedness in mediating the effect of physical/functional limitations on loneliness. Items from validated instruments were used to measure four latent variables (physical/functional limitations, resilience, loneliness and social connectedness). Structural parallel mediator models estimated each path in the mediation analysis controlling for gender, education, and age. Confidence intervals for mediation effects were generated via bias-corrected bootstrapping with 10,000 replications. The total effect of physical/functional limitations on perceived loneliness was significant. Social connectedness and resilience fully and significantly mediated the relationship between physical/functional limitations and loneliness. The indirect effect (B =.143; 95% CI = .047, .280) for the physical/functional limitations-resilience-loneliness pathway indicated that the positive effect of physical/functional limitations on loneliness was approximately 0.143 points lower as mediated by higher resilience. The indirect path of physical/functional limitations on loneliness through the mediation of social connectedness (B =.077; 95% CI = .010, .168) yielded a reduction of .077 points. The total amount of variance in feelings of loneliness accounted for by the overall model, which included the proposed mediators and control variables, was 48.6%. Interventions to build resilience and social interactions may attenuate the effects of physical/functional disability and loneliness on health outcomes in individuals at risk.

